# A Molecular Candle Where Few Molecules Shine: HeHHe^+^

**DOI:** 10.3390/molecules25092183

**Published:** 2020-05-07

**Authors:** Ryan C. Fortenberry, Laurent Wiesenfeld

**Affiliations:** 1 Department of Chemistry & Biochemistry, University of Mississippi, University, MS 38677-1848, USA; 2 Laboratoire Aimé-Cotton, CNRS & Université Paris-Saclay, 91405 Orsay, France; laurent.wiesenfeld@u-psud.fr

**Keywords:** helium chemistry, infrared spectroscopy, early universe, quantum chemistry

## Abstract

HeHHe${}^{+}$ is the only potential molecule comprised of atoms present in the early universe that is also easily observable in the infrared. This molecule has been known to exist in mass spectrometry experiments for nearly half-a-century and is likely present, but as-of-yet unconfirmed, in cold plasmas. There can exist only a handful of plausible primordial molecules in the epochs before metals (elements with nuclei heavier than ${}^{4}$He as astronomers call them) were synthesized in the universe, and most of these are both rotationally and vibrationally dark. The current work brings HeHHe${}^{+}$ into the discussion as a possible (and potentially only) molecular candle for probing high-*z* and any metal-deprived regions due to its exceptionally bright infrared feature previously predicted to lie at 7.43 μm. Furthermore, the present study provides new insights into its possible formation mechanisms as well as marked stability, along with the decisive role of anharmonic zero-point energies. A new entrance pathway is proposed through the triplet state (${}^{3}B_{1}$) of the He${}_{2}$H${}^{+}$ molecule complexed with a hydrogen atom and a subsequent 10.90 eV charge transfer/photon emission into the linear and vibrationally-bright ${}^{1}\Sigma_{g}^{+}$ HeHHe${}^{+}$ form.

## 1. Introduction

Atomic and molecular spectroscopic signatures are the fingerprints of the universe. In the era before metals (metals in the astrophysical acceptance are atoms built with nuclei heavier than ${}^{4}$He), only ${}^{1}$H and ${}^{4}$He, with a trace amount of lithium (along with ${}^{2}$H and ${}^{3}$He), comprised the matter of the universe. The richness of chemistry would only come later when stars could turn these nuclei into carbon, nitrogen, oxygen, and the rest of the periodic table. Hence, observations of the early universe have to rely upon species comprised of these first two elements (leaving ${}^{6,7}$Li out for now). For the high-*z* universe, see a vivid example in Ref. [1] or a general review in Ref. [2]. Also, some objects in space are very metal poor and are considered as relics of very old stars and galaxies [3]. Lastly, in molecular gases subjected to intense X-ray exposure, especially so in Active Galactic Nuclei [4], chemistry is profoundly modified because of the ionization of both hydrogen and helium. Helium is the most costly atom to ionize, with $E_{\mathrm{ionization}}≃24.587\;\mathrm{eV}$. The presence of the highly reactive He${}^{+}$, He${}_{2}^{+}$ ions is therefore a driver of such primordial chemistry, along with H${}_{2}^{+}$ and H${}_{3}^{+}$ [5,6].

The helonium molecule (HeH${}^{+}$) is the quintessential example for chemistry involving no metal atoms. This diatomic species was first observed in the laboratory nearly a century ago [7] but was only recently detected in nature in NGC 7027 [8,9]. Unfortunately, astronomical surveys in search of this diatomic molecule at the very edge of the universe have come up blank [10] with the most likely explanation that the spectroscopic features of HeH${}^{+}$ are too dim to be observed at such high redshift. Such an explanation still does not seem to satisfy fully the scientific community.

Differently, the helium diatomic cation (He${}_{2}^{+}$) is a surprising participant in the chemistry of the primordial epoch [5]. He${}_{2}^{+}$ is known to possess a fairly strong bond energy of roughly half that of the O−H bond in water [11]. However, a lack of dipole moment or even an induced dipole preclude its observation, even more so than the hydrogen molecule. H${}_{2}$, on the other hand, is observed in absorption against background intense light sources thanks to its high abundance and its weak quadrupolar electric (E2) transition at 28.27 μm [12]. Consequently, another molecule must be utilized to trace observations of high-*z* and/or metal poor regions.

While there are a surprising number of possible hydro-helium species [11,13,14], none are as tantalizing as the triatomic molecule, HeHHe${}^{+}$. Even if this molecule also has no permanent dipole moment precluding any rotational observation, it has recently been shown quantum chemically to possess an exceptionally bright vibrational frequency (the antisymmetric stretch) at 1345.2 cm${}^{-1}$/7.43 μm [15]. In fact, the astrophysical detection of the related HeH${}^{+}$ molecule was actually done in the infrared even if for a rotational signature [8]. The intensity of this HeHHe${}^{+}$ band is on the order of forty times brighter than that of the “bright” antisymmetric stretch of water, and there is a second band at 884.9 cm${}^{-1}$/11.3 μm (the π-bending fundamental) with an intensity four times brighter than that of the same frequency in water. Hence, HeHHe${}^{+}$ is a possible target for astronomical observations especially at high-*z*.

HeHHe${}^{+}$ has been known in mass spectrometry experiments since 1970 with many subsequent reportings since [16,17,18]. The related ArHAr${}^{+}$ molecule has been spectroscopically classified experimentally and quantum chemically [19,20], but no experimental electronic or vibrational spectra yet exist for the helium analogue in spite of recent theoretical work [21]. Even so, the quantum chemical data [15,21] show that this molecule, even at fairly low abundances, should be detectable. Terrestrially, HeHHe${}^{+}$ should exist and be observable in cold plasmas. Such experiments will be key in classifying this molecule for astronomical observation. Questions still remain as to how HeHHe${}^{+}$ can form or persist in the gas phase even for the relatively warm and dense conditions of the high-*z* universe or in the presence of abundant H atoms and H${}_{2}$ molecules.

The formation of HeHHe${}^{+}$ has been suggested to take place through reactions of He${}_{2}$${}^{+}$ with H${}_{2}$ giving off a hydrogen atom [16], and a major product is the linear and centro-symmetric HeHHe${}^{+}$ molecule in addition to other species such as HeH${}^{+}$ and HeH${}_{2}$${}^{+}$ [22,23,24,25,26]. The triatomic structure has been shown to be well-bound in these studies, but pathways for the creation of this molecule suffer either from states that are too deep in the wells of their potential energy surfaces (PESs) or that are quasi- or completely unbound [24]. Previous quantum chemical work claims that the ${}^{1}\Sigma_{g}^{+}$ ground state of HeHHe${}^{+}$ is the most-strongly bound state with an additional, weakly-bound ${}^{1}A_{1}$ isosceles triangle structure also present [23]. This same previous study does not report any bound triplet states in linear or triangular/cyclic isomers. The present study will give an updated reporting on the singlet and triplet PESs for [He${}_{2}$, H]${}^{+}$ and comment on the potential role that HeHHe${}^{+}$ can have in tracing primordial gas.

HeHHe${}^{+}$ is of great chemical relevance, but its thermodynamic properties should be also considered in detail, especially for the role that it could play in the primordial universe. The cooling of matter is an issue of paramount importance in order to structure the early universe. To form the very first stars in the absence of metals (the so-called population III stars), both macrophysics and microphysics have to be constrained with a very close interplay between both. The gravitationally imploding gas heats up because of conversions of the gravitational potential energy into kinetic energy, and, then, the gas has to cool in order for stars to form. Previously, H${}_{2}$ was thought to be the principal coolant of gas yielding to population III stars [27], but more recent astrophysical stellar computational models have opened the landscape to very minor components, albeit with much more favorable radiative properties potentially including HeHHe${}^{+}$.

In Galli [5] (Figure 5), the role of the extremely minor species LiH, H${}_{3}^{+}$, and HD largely supersedes the H${}_{2}$ cooling power by 6 to 14 orders of magnitudes, for T≤ of a few hundred K [28]. This conclusion is however still under debate [29], and precise computations either in local thermodynamical equilibrium (LTE) [28] or non-LTE conditions might be necessary [30]. Note that for species undergoing radiative spontaneous emission, cooling rate computations are much easier than collisional cooling with the only ingredient being the *A* Einstein coefficient for the transition. Since the HeHHe${}^{+}$ molecule possesses a very large transition dipole in the IR, its role in the cooling of metal-poor gases cannot be neglected off-handedly and such an intense molecular radiator is of crucial importance to model the few ingredients that will constrain the initial mass function of the population III stars.

## 2. Computational Detals

The “gold standard of quantum chemistry”, coupled cluster theory at the singles, doubles, and perturbative triples [CCSD(T)] level [31,32,33], is utilized in this work but in the more complete explicitly correlated (F12b) formalism [34,35] with an aug-cc-pVTZ basis set [36,37,38]. The relative energies are computed from the energies of the CCSD(T)-F12/aug-cc-pVTZ optimized geometries. CCSD(T)-F12 at the triple-ζ basis level has recently been shown to produce vibrational frequencies often within less than 5 cm${}^{-1}$ of much higher levels of theory including considerations made for complete basis set extrapolations, scalar relativity, and core electron correlation [39,40,41]. For these molecules, relativity for the electronic structure will be insignificant, and there are no core electrons. The F12 formalism is known to provide more electron correlation for smaller basis sets and thus notable accuracy even for triple-ζ basis sets when compared to the complete basis set values [42,43].

The harmonic frequencies are then computed, and the subsequent zero-point vibrational energy (ZPVE) is then added to the energy of each molecule or atom. One can read that the simple products minus reactants chemical energy computations produce the relative energy values that are collected and discussed later. The PESs are computed by displacements of 0.1 Å for any bond length coordinates and 2.0${}^{\circ }$ for any bond angles. The two-dimensional PES scans have the coordinates transformed into planar Cartesian ones for the positions of the respective H and He atoms. In all cases, the energies are then made relative to the minima of each PES.

## 3. Results

**Figure 1:**
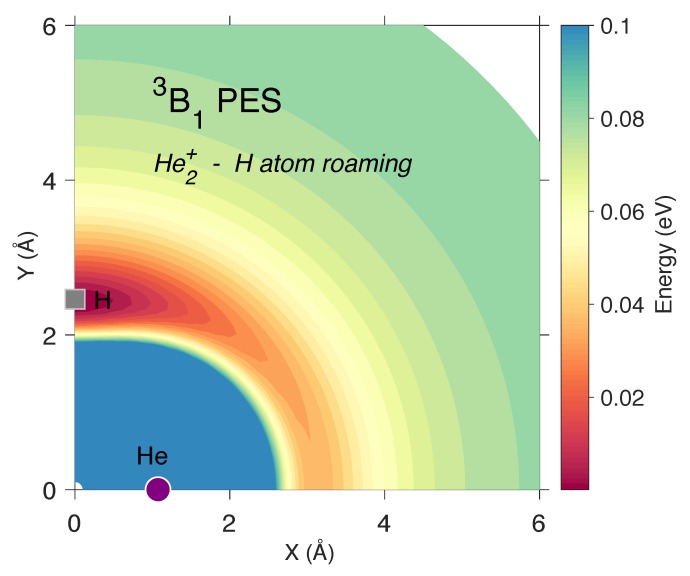
The two-dimensional triplet He${}_{2}$H${}^{+}$ PES in eV for the motion of the hydrogen atom (gray square) around the fixed positions of the helium atoms (purple circles) in the ${}^{2}\Sigma_{u}$ He${}_{2}$${}^{+}$ molecule with one shown on the x-axis. The minimum corresponds to the geometry of the ${}^{3}B_{1}$ state, 10.90 eV above the minimum of the ground ${}^{1}\Sigma_{g}^{+}$ state.

The present work shows that several bound states have been missed for this molecule and could play an important role in its formation or persistence in the metal-deprived universe. Most notably, this study shows that the [He${}_{2}$, H]${}^{+}$ PES actually possesses a bound triplet state in an isosceles triangle arrangement that could serve as an entrance channel for the creation of the more stable and linear ${}^{1}\Sigma_{g}^{+}$ ground state of HeHHe${}^{+}$. This ${}^{3}B_{1}$ He${}_{2}$H${}^{+}$ molecule has a well-defined minimum that requires 0.053 eV to remove the hydrogen atom leading to ${}^{2}\Sigma_{u}$ He${}_{2}$${}^{+}$ and H${(}^{2}S_{\frac{1}{2}})$. While the bond dissociation energy is small, it is less than the fundamental vibrational frequency for the hydrogen stretching mode ($\nu_{3}$) at 115.5 cm${}^{-1}$ or 0.014 eV. The two-dimensional plot of the hydrogen atom roaming (Figure 1) shows that the isosceles triangle ($C_{2v}$) structure is the minimum with a van der Waals ring encircling the helium dimer cation.

${}^{3}B_{1}$ He${}_{2}$H${}^{+}$ will also be rotationally active due to its atomic arrangement and modest dipole moment (0.83 D), and the full set of rotational constants and vibrational frequencies are given in Table 1. Benchmarks for CCSD(T)-F12/aug-cc-pVTZ for He${}_{2}$${}^{+}$ compared with derived experimental data [44] are quite good in accordance with recent vibrational benchmarks for this method [39,40,41]. Furthermore, the CCSD(T)-F12/aug-cc-pVTZ values are nearly identical to those computed with the composite method utilized previously [15]. The principal rotational constant for ${}^{3}B_{1}$ He${}_{2}$H${}^{+}$ is nearly the same as that for He${}_{2}$${}^{+}$ since the He−He bond lengths are also very closely aligned, and the additional hydrogen atom’s relatively small mass lies on the symmetry axis of the system. The $\nu_{1}$ He−He stretching fundamentals of the ${}^{3}B_{1}$ He${}_{2}$H${}^{+}$ and He${}_{2}$${}^{+}$ are also close to degenerate, but this mode has a very small intensity for the triatomic and none by symmetry for the diatomic. As a result, any potential detections of ${}^{3}B_{1}$ He${}_{2}$H${}^{+}$ and its possible role in the formation of ${}^{1}\Sigma_{g}^{+}$ HeHHe${}^{+}$ will likely have to come from rotational spectroscopy and not vibrational/IR spectroscopy.

A simple Mulliken population analysis or molecular orbital plotting of ${}^{3}B_{1}$ He${}_{2}$H${}^{+}$ shows that the hydrogen atom is neutral, and the two helium atoms evenly share 50% of the remaining positive charge. This and the relatively weak binding indicate that ${}^{3}B_{1}$ He${}_{2}$H${}^{+}$ is a van der Waals complex of He${}_{2}$${}^{+}$ and a hydrogen atom. However, the shallow well would allow for relatively fast radiative association through this pathway. Then, the system could emit a photon and collapse down to the singlet PES. This would basically be a charge transfer more than a spin-flip electronic emission into the singlet state where the positive charge would rest solely on the hydrogen atom in the singlet instead of being shared by the two helium atoms in the triplet. Such a charge transfer and relaxation from the triplet to the singlet PES should be fast since the ionization of a hydrogen atom (13.6 eV) is significantly less than that of a helium atom at 24.6 eV which is the highest atomic ionization potential.

**Figure 2:**
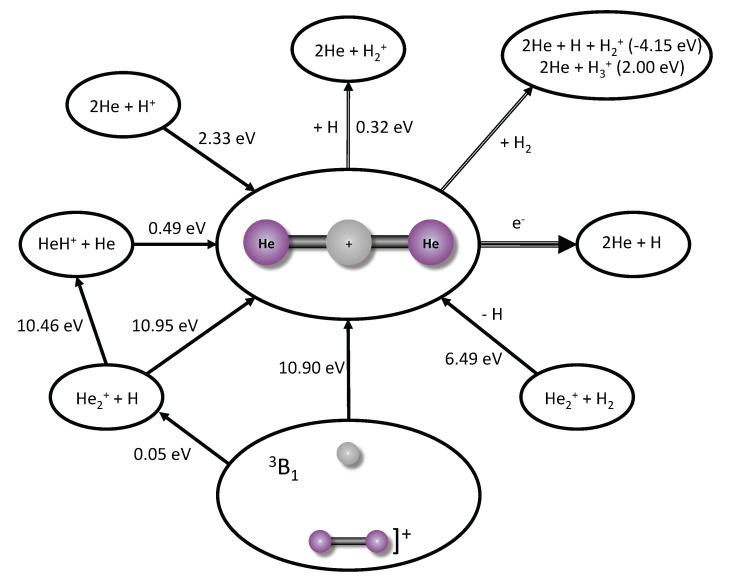
The pathways and ZPVE-corrected energetics for the creation and destruction of ${}^{1}\Sigma_{g}^{+}$ HeHHe${}^{+}$. Positive energies favor the products based on the direction of the arrow. Negative energies favor the reactants.

Figures S1 and S2 in the supplemental information (SI) provide the two-dimensional descriptions of the [He${}_{2}$, H]${}^{+}$ PES for both the singlet and the triplet spin states with Jacobi coordinates of the H−X (where X is the center of mass between the two helium atoms) and He−He bond lengths. These figures have *∠*H−X−He fixed at 90.0${}^{\circ }$ in line with the minimum from Figure 1. Adiabatically accounting for the ZPVE, the difference in the minima of the ${}^{3}B_{1}$ and ${}^{1}\Sigma_{g}^{+}$ states is 10.90 eV as shown in the bottom of Figure 2. However, the vertical or Franck-Condon (non-ZPVE) difference is 6.70 eV (185 nm) from the triplet minimum to the singlet PES. It must be underlined on the one hand that this transition being a triplet → singlet one, and with only *s* electrons present, it would be considerably slower (by at least a factor $\alpha^{2}$, α≃1/137, the fine-structure constant) than its usual electric dipole counterpart. Franck-Condon factors, on the other hand, could be sizeable due to the highly unlocalized eigenfunctions, signalled by the high ZPVEs.

Regardless of the process, the singlet structure could relax down the PES where the helium atoms would further separate from a distance (given in Table 1) of 1.074 Å to 1.893 Å. The hydrogen atom would then migrate from lying 2.615 Å away from the center-of-mass to resting at the center-of-mass. The dynamics of this action are beyond the scope of the current work, but Figures S1 and S2 show that once the electronic relaxation/charge transfer from the triplet to the singlet state takes place, the required atomic movements into the centrosymmetric linear form are barrierless. Furthermore, such a motion would certainly vibrationally excite the π-bending frequency at 884.9 cm${}^{-1}$/11.3 μm which is also fairly bright making this mode and its vibrational cascade during these dynamics also targets for detection of HeHHe${}^{+}$.

Differently, vertically moving from the ${}^{1}\Sigma_{g}^{+}$ HeHHe${}^{+}$ ground state minimum onto the triplet PES would require input of 20.66 eV (60 nm) largely due to the repulsive nature of the triplet PES in the region where the hydrogen atom separates the helium atoms. Such energetics all but ensure that once the singlet surface is accessed, it will remain locked in this spin configuration until the point of dissociation.

Dissociation of the ${}^{1}\Sigma_{g}^{+}$ HeHHe${}^{+}$ molecule will also not be spontaneous. Removal of a single helium atom requires 0.49 eV (shown on the left of Figure 2) in line with such values computed previously [15,22,25,26] and notably *more than* any of the ν=1 or even ν=2 vibrational states. Total atomization requires 2.33 eV. Destruction of ${}^{1}\Sigma_{g}^{+}$ HeHHe${}^{+}$ will most readily take place through electron insertion (dissociative recombination) where neutrals are created of all the species and the relative energy given off will largely depend upon the kinetic energy of the incident electron. Insertion of a proton will be Coulombically disfavored, but insertion of a second hydrogen atom will produce two helium atoms and H${}_{2}$${}^{+}$. While this will produce 0.32 eV of energy thermodynamically, preliminary computations show a barrier to this process which could slow the kinetics. This will be explored in future work. Finally, in Figure 2 addition of H${}_{2}$ to ${}^{1}\Sigma_{g}^{+}$ HeHHe${}^{+}$ thermodynamically disfavors charge transfer in the hydrogen molecule and HeHHe${}^{+}$ total atomization but does favor creation of H${}_{3}$${}^{+}$ and two helium atoms.

**Figure 3:**
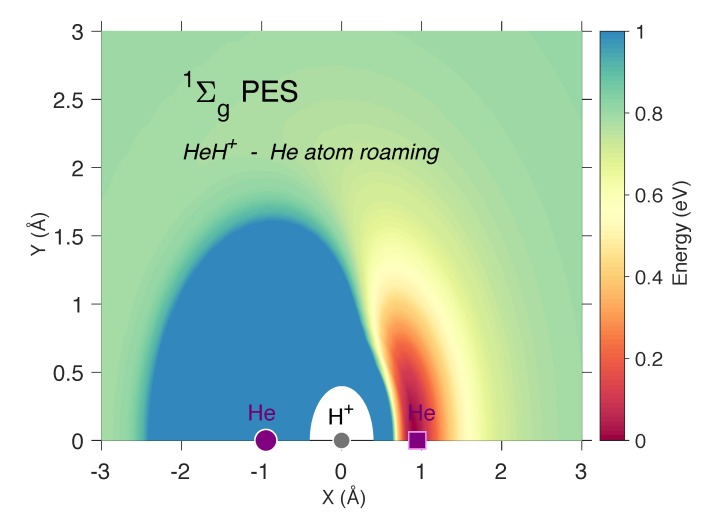
The two-dimensional Singlet HeH${}^{+}$ + He PES in eV for the motion of the helium atom (purple square) around the fixed positions of the hydrogen and other helium atom (gray and purple circles, respectively) in the ${}^{1}\Sigma_{g}^{+}$ HeHHe${}^{+}$ molecule. The minimum corresponds to the geometry of the ${}^{1}\Sigma_{g}^{+}$ state.

Additionally, the singlet [He${}_{2}$, H]${}^{+}$ PES produces a previously unknown van der Waals minimum for association of a helium atom encircling the HeH${}^{+}$ cation. While the association of a single helium atom with HeH${}^{+}$ is probable [26], the most likely product is simply transference of the proton with the orginal helium atom translating away a majority of the kinetic energy. However, in dense enough regions like the warm primordial medium or in cold plasmas, this additional pathway could allow for some association. While the PES for the motion of a single helium atom around HeH${}^{+}$ given in Figure 3 is nearly identical to previous formulations highlighting the consistency of behavior for this molecule [23,24,25,26], a small van der Waals minimum is present outside the current x-range but given in Figure S3. The energy dips slightly when the two helium nuclei are adjacent to one another, collinear with the helonium, and 2.103 Å apart. Granted, the minimum is a mere 0.012 eV below the dissociation limit, but the zero-point energy for the He−He stretch in this complex is 0.011 eV indicating that it will not necessarily spontaneously dissociate even though the minimum will be rather shallow. Hence, a new channel for HeH${}^{+}$ + He association may be present.

## 4. Discussion and Conclusions

In the era of the universe before nuclear synthesis and in metal-deprived regions of the cosmos, molecules can really only be comprised of hydrogen and helium. Consequently, molecular spectral absorption/emission lines, the veritable fingerprint of the cosmos, must depend upon such molecular species. The diatomic species H${}_{2}$, H${}_{2}^{+}$, and He${}_{2}$${}^{+}$ are believed to be present but are virtually untraceable. Isotopic substitution is possible, but the resulting dipoles or induced dipoles are very weak and would be quite difficult to observe in the necessary high-*z* regions. HeH${}^{+}$ has been a tantalizing molecule for tracing such an environment, but it has escaped observation for unknown reasons.

The triatomic species of this time must all be charged. H${}_{3}^{+}$ is modelled to be common, but it is also a weak spectral sentinel since it has no dipole moment and a small vibrationally-induced dipole making its infrared transitions exceedingly weak. HeH${}_{2}$${}^{+}$ may also be viable, but preliminary computations put the He + H${}_{2}$${}^{+}$ dissociation at only 0.21 eV above the minimum making its persistence unlikely in hot environments or regions with high collision rates. Furthermore, the tetraatomic HeH${}_{3}$${}^{+}$ is even more weakly bound [14,15]. Therefore, HeHHe${}^{+}$ is the most viable molecular tracer of the metal-poor universe.

This work shows that in the epochs or locations where He${}_{2}$${}^{+}$ is present, an incident hydrogen atom could associate through the ${}^{3}B_{1}$ state of He${}_{2}$H${}^{+}$ before a 10.90 eV ultraviolet charge transfer/electronic emission to the singlet state creating the ${}^{1}\Sigma_{g}^{+}$ HeHHe${}^{+}$ form after energetically favored atomic rearrangement. Upon its creation, ${}^{1}\Sigma_{g}^{+}$ HeHHe${}^{+}$ should persist since it is impervious to thermal radiation below 5700 K and to photons from the near-IR or longer wavelengths. Unless there is a collision with an electron or high-velocity hydrogen atom or molecule, the near-IR dissociation of ${}^{1}\Sigma_{g}^{+}$ HeHHe${}^{+}$ into helonium and a hydrogen atom could potentially recombine into the triatomic again from the van der Waals minimum. This recombination could also take place for helonium in the presence of helium atoms. Such would explain why HeH${}^{+}$ is not visible beyond its spectroscopic properties. Helonium would form into HeHHe${}^{+}$ or dissociate into helium atoms and various charged forms of hydrogen atoms and molecules depending upon the colliding particle. Dynamics of the HeHHe${}^{+}$ complex depends intimately on the ZPVE as well as the construction of the eigenstates. The resulting transition rates will be an example of fully quantum-dominated dynamics.

Finally, the proton shuttling motion in ${}^{1}\Sigma_{g}^{+}$ HeHHe${}^{+}$ is an exceptionally strong emitter/absorber at 7.435 μm (not corrected for *z*). In the epochs where this molecule might form, this bright feature will likely be observable over a spectral swathe ranging from microwaves to the far-IR. Similar behavior is also possible for the bright bending motion at 11.3 μm. Hence, either of these features could be among the very earliest detectable molecular signatures and would serve as a candle for observations of such regions on the edge of the visible universe. Refinement of the vibrational frequency is needed from experiment, and cold helium plasmas are the best place to search for these spectral features in laboratory measurements. 

## Figures and Tables

**Table 1:** The CCSD(T)-F12/aug-cc-pVTZ Zero-Point ($R_{\alpha }$) Geometries, Vibrational Frequencies (Intensities ${}^{a}$ in Parentheses), and Spectroscopic Constants for ${}^{3}B_{1}$ He${}_{2}$H${}^{+}$, ${}^{2}\Sigma_{u}$ He${}_{2}$${}^{+}$, and ${}^{1}\Sigma_{g}^{+}$ HeHHe${}^{+}$.

		${}^{2}\Sigma_{u}$He${}_{2}$${}^{+}$		
	${}^{3}B_{1}$He${}_{2}$H${}^{+}$	This Work	Exp. ${}^{b}$	${}^{1}\Sigma_{g}^{+}$HeHHe${}^{+}$
r${}_{0}$(He−He) Å	1.073 750	1.076 016	1.080	1.892 704
r${}_{0}$(H−X ${}^{c}$) Å	2.615 334			0.946 352
$A_{0}$ GHz	227.185			
$B_{0}$ GHz	86.893	226.228	216.2	70.837
$C_{0}$ GHz	60.225			
μ D	0.83			
$\omega_{1}$ cm${}^{-1}$	1706.4 (1)	1698.8	1698.5	1554.7 (2661)
$\omega_{2}$ cm${}^{-1}$	324.1 (22)			955.1 (294)
$\omega_{3}$ cm${}^{-1}$	186.4 (2)			1139.5
Zero-Point cm${}^{-1}$	1074.8	831.7		2261.4
$\nu_{1}$ cm${}^{-1}$	1632.6	1625.0		1350.6
$\nu_{2}$ cm${}^{-1}$	269.2			889.8
$\nu_{3}$ cm${}^{-1}$	115.5			896.0
$2\nu_{3}$ cm${}^{-1}$	136.1			

${}^{a}$ The double-harmonic intensities (in km/mol) and dipole moments are from MP2/aug-cc-pVDZ. The intensities for ${}^{1}\Sigma_{g}^{+}$ HeHHe${}^{+}$ are from previous computations [15]; ${}^{b}$ Experimental results from extrapolations of ${}^{3}\Pi_{g}$ He${}_{2}$ [44]; ${}^{c}$ X is the center of mass between the two helium atoms in ${}^{3}B_{1}$ He${}_{2}$H${}^{+}$, and X is He in ${}^{1}\Sigma_{g}^{+}$ HeHHe${}^{+}$.

## Supplementary Materials

The following are available online, Figure S1: PES in Jacobi coordinates for the singlet ground state of HeHHe${}^{+}$, Figure S2: PES in Jacobi coordinates for the triplet first excited state of HeHHe${}^{+}$, Figure S3: Van der Waals minimum on the singlet surface. Technical points of the quantum chemistry employed are also provided.
