# Supplementary material for: A Molecular Candle Where Few Molecules Shine: HeHHe+
Source: Molecules. 2020 May 7;25(9):2183. doi: 10.3390/molecules25092183 (PMC7249080; doi:10.3390/molecules25092183)
Supplement: Supplementary file 1 [file molecules-25-02183-s001.pdf]

Supplementary Information for A Molecular Candle where Few Molecules Shine: $HeHHe^+$

Ryan C. Fortenberry[1,*] and Laurent Wiesenfeld[2]

[1]*Department of Chemistry & Biochemistry,*

University of Mississippi, University, Mississippi 38677, U.S.A.

[2]*Laboratoire Aimé-Cotton, Université Paris-Saclay and CNRS, Orsay, France*

(Dated: April 8, 2020)

QUANTUM CHEMISTRY METHODS

The CCSD(T)-F12/aug-cc-pVTZ computations utilized here are computed with the MOLPRO 2015.1 quantum chemistry program.[1] The anharmonic frequencies are computed via a fourth-order Taylor series expansion of the portion within the internuclear Hamiltonian, a quartic force field or QFF.[2] Such explicitly correlated QFFs have shown promise in producing closely comparable data to more advanced QFFs[3, 4] and even experiment for fractions of the computational cost.[5, 6] The displacements for the $^3B_1$ He_2H^+ geometry follow those from other C_{2v} triatomics,[7, 8] and those for $^1\Sigma_g$ $HeHHe^+$ are the same from the previous work.[9] In either case, the force constants are fit via a least squares approach and refit to give zero gradients. The force constants are transformed into Cartesian coordinates in the INTDER program,[10] and fed into vibrational perturbation theory at second-order (VPT)[11–13] in the SPECTRO program.[14] The double-harmonic vibrational intensities are computed with MP2/aug-cc-pVDZ in the Gaussian09 program.[15, 16]

The relative energies are computed from the energies of the CCSD(T)-F12/aug-cc-pVTZ optimized geometries. The harmonic frequencies are then computed, and the subsequent ZPVE is then added to the energy of each molecule or atom. The one exception are the anharmonic frequencies included in the discussion surrounding Figure 1 in the main text. Then, simple products minus reactants chemical energy computations produce the relative energy values that are collected in Figure 3.

The PESs are computed by displacements of 0.1 Å for any bond length coordinates and 2.0° for any bond angles. Figures 3 and 4 have the coordinates transformed into planar Cartesian positions (x & y) for the positions of the respective H and He atoms. In all cases, the energies are then made relative to the minima of each PES.

FIGURES

Figures SI and SII represent respectively the singlet $^1\Sigma_g$ and triplet $^3B_1$ PES, in Jacobi coordinates. If X is the center of massof the He_2^+ fragment, R is the XH distance, r the He_2^+ bond length and θ is the angle berween the $\mathbf{r}$ and $\mathbf{R}$ vectors. Note that the origin of energy is different between Figures SI and SII; the triplet minimum is $\Delta E_O = 11.13$ eV above the singlet state minimum, in the non-ZPVE picture. Figure SIII is a one-D cut of the singlet

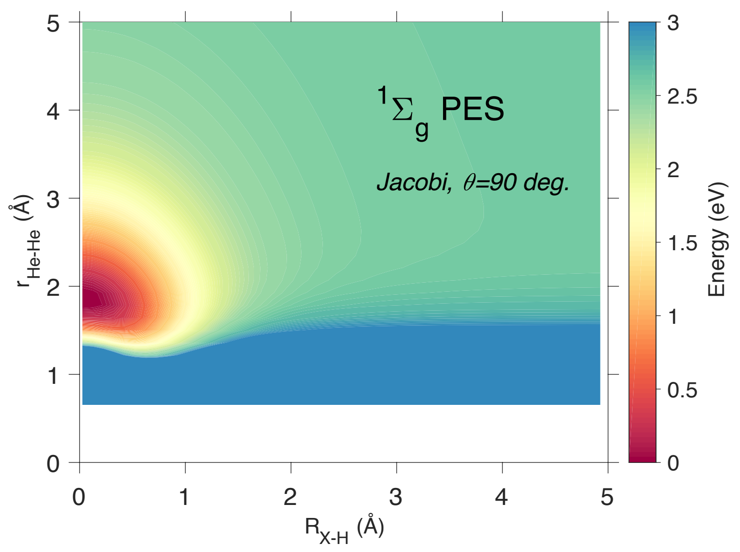

FIG. SI. PES in Jacobi coordinates for the singlet ground state of $HeHHe^+$.

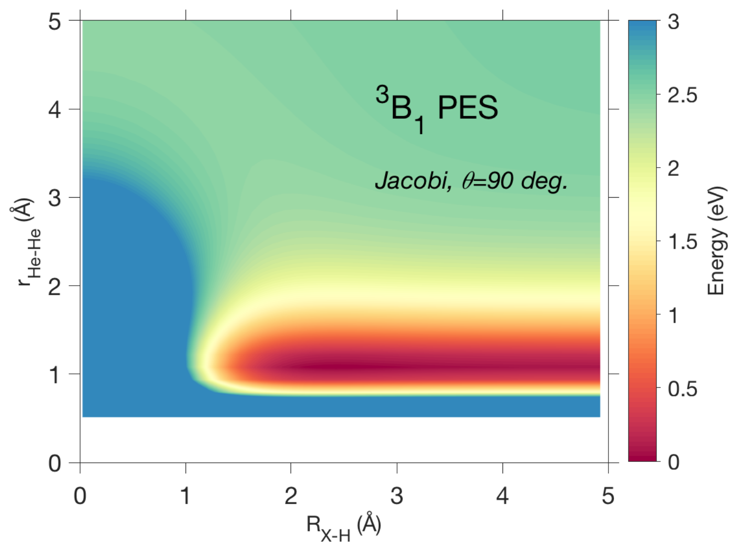

FIG. SII. PES in Jacobi coordinates for the triplet first excited state of $HeHHe^+$.

surface, along the X direction, showing the van der Waals minimum at large distance.

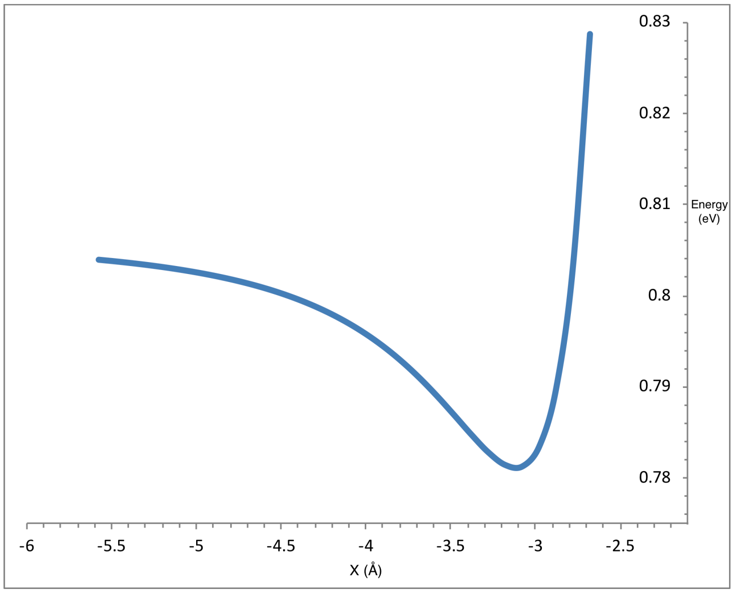

FIG. SIII. Van der Waals minimum on the singlet surface relative to the global energy minimum: A one-D cut of the PES for He drifting away from helonium HeH^+.

* r410@olemiss.edu

[1] H.-J. Werner, P. J. Knowles, G. Knizia, F. R. Manby, M. Schütz, P. Celani, W. Györffy, D. Kats, T. Korona, R. Lindh, A. Mitrushenkov, G. Rauhut, K. R. Shamasundar, T. B. Adler, R. D. Amos, A. Bernhardsson, A. Berning, D. L. Cooper, M. J. O. Deegan, A. J. Dobbyn, F. Eckert, E. Goll, C. Hampel, A. Hesselmann, G. Hetzer, T. Hrenar, G. Jansen, C. Köppl, Y. Liu, A. W. Lloyd, R. A. Mata, A. J. May, S. J. McNicholas, W. Meyer, M. E. Mura, A. Nicklass, D. P. O'Neill, P. Palmieri, D. Peng, K. Pflüger, R. Pitzer, M. Reiher, T. Shiozaki, H. Stoll, A. J. Stone, R. Tarroni, T. Thorsteinsson, and M. Wang, "Molpro, version 2015.1, a package of *ab initio* programs," (2015), see http://www.molpro.net.

[2] X. Huang and T. J. Lee, J. Chem. Phys. **129**, 044312 (2008).

[3] X. Huang, P. R. Taylor, and T. J. Lee, J. Phys. Chem. A **115**, 5005 (2011).

[4] R. C. Fortenberry, X. Huang, J. S. Francisco, T. D. Crawford, and T. J. Lee, J. Chem. Phys. **135**, 134301 (2011).

[5] R. C. Fortenberry, T. Trabelsi, and J. S. Francisco, J. Phys. Chem. A **122**, 4983 (2018).

[6] C. Z. Palmer and R. C. Fortenberry, J. Phys. Chem. A (2018), *submitted*.

[7] R. C. Fortenberry, T. J. Lee, and H. S. P. Müller, Molec. Astrophys. **1**, 13 (2015).

[8] R. C. Fortenberry, R. Thackston, J. S. Francisco, and T. J. Lee, J. Chem. Phys. **147**, 074303 (2017).

[9] C. J. Stephan and R. C. Fortenberry, Mon. Not. Royal Astron. Soc. **469**, 339 (2017).

[10] W. D. Allen and coworkers, (2005), *INTDER* 2005 is a General Program Written by W. D. Allen and Coworkers, which Performs Vibrational Analysis and Higher-Order Non-Linear Transformations.

[11] I. M. Mills, in *Molecular Spectroscopy - Modern Research*, edited by K. N. Rao and C. W. Mathews (Academic Press, New York, 1972) pp. 115–140.

[12] J. K. G. Watson, in *Vibrational Spectra and Structure*, edited by J. R. During (Elsevier, Amsterdam, 1977) pp. 1–89.

[13] D. Papousek and M. R. Aliev, *Molecular Vibration-Rotation Spectra* (Elsevier, Amsterdam, 1982).

[14] J. F. Gaw, A. Willets, W. H. Green, and N. C. Handy, in *Advances in Molecular Vibrations and Collision Dynamics*, edited by J. M. Bowman and M. A. Ratner (JAI Press, Inc., Greenwich, Connecticut, 1991) pp. 170–185.

[15] C. Møller and M. S. Plesset, Phys. Rev. **46**, 618 (1934).

[16] M. J. Frisch, G. W. Trucks, H. B. Schlegel, G. E. Scuseria, M. A. Robb, J. R. Cheeseman, G. Scalmani, V. Barone, B. Mennucci, G. A. Petersson, H. Nakatsuji, M. Caricato, X. Li, H. P. Hratchian, A. F. Izmaylov, J. Bloino, G. Zheng, J. L. Sonnenberg, M. Hada, M. Ehara, K. Toyota, R. Fukuda, J. Hasegawa, M. Ishida, T. Nakajima, Y. Honda, O. Kitao, H. Nakai, T. Vreven, J. A. Montgomery, Jr., J. E. Peralta, F. Ogliaro, M. Bearpark, J. J. Heyd, E. Brothers, K. N. Kudin, V. N. Staroverov, R. Kobayashi, J. Normand, K. Raghavachari, A. Rendell, J. C. Burant, S. S. Iyengar, J. Tomasi, M. Cossi, N. Rega, J. M. Millam, M. Klene, J. E. Knox, J. B. Cross, V. Bakken, C. Adamo, J. Jaramillo, R. Gomperts, R. E. Stratmann, O. Yazyev, A. J. Austin, R. Cammi, C. Pomelli, J. W. Ochterski, R. L. Martin, K. Morokuma, V. G. Zakrzewski, G. A. Voth, P. Salvador, J. J. Dannenberg, S. Dapprich, A. D. Daniels, O. Farkas, J. B. Foresman, J. V. Ortiz, J. Cioslowski, and D. J. Fox, "Gaussian 09 Revision D.01," (2009), gaussian Inc. Wallingford CT.
